# Leveraging medical context to recommend semantically similar terms for chart reviews

**DOI:** 10.1186/s12911-021-01724-2

**Published:** 2021-12-18

**Authors:** Cheng Ye, Bradley A. Malin, Daniel Fabbri

**Affiliations:** 1 Department of Computer Science, Vanderbilt University, 2301 Vanderbilt Place, PMB 351679, Nashville, TN 37235-1679 USA; 2 Department of Biomedical Informatics, Vanderbilt University Medical Center, Nashville, TN USA; 3 Department of Biostatistics, Vanderbilt University Medical Center, Nashville, TN USA

**Keywords:** Electronic medical records, Data science, Chart reviews, Clinically similar terms, Vector space model

## Abstract

**Background:**

Information retrieval (IR) help clinicians answer questions posed to large collections of electronic medical records (EMRs), such as how best to identify a patient’s cancer stage. One of the more promising approaches to IR for EMRs is to expand a keyword query with similar terms (e.g., augmenting *cancer* with *mets*). However, there is a large range of clinical chart review tasks, such that fixed sets of similar terms is insufficient. Current language models, such as Bidirectional Encoder Representations from Transformers (BERT) embeddings, do not capture the full non-textual context of a task. In this study, we present new methods that provide similar terms dynamically by adjusting with the context of the chart review task.

**Methods:**

We introduce a vector space for medical-context in which each word is represented by a vector that captures the word’s usage in different medical contexts (e.g., how frequently *cancer* is used when ordering a prescription versus describing family history) beyond the context learned from the surrounding text. These vectors are transformed into a vector space for customizing the set of similar terms selected for different chart review tasks. We evaluate the vector space model with multiple chart review tasks, in which supervised machine learning models learn to predict the preferred terms of clinically knowledgeable reviewers. To quantify the usefulness of the predicted similar terms to a baseline of standard word2vec embeddings, we measure (1) the prediction performance of the medical-context vector space model using the area under the receiver operating characteristic curve (AUROC) and (2) the labeling effort required to train the models.

**Results:**

The vector space outperformed the baseline word2vec embeddings in all three chart review tasks with an average AUROC of 0.80 versus 0.66, respectively. Additionally, the medical-context vector space significantly reduced the number of labels required to learn and predict the preferred similar terms of reviewers. Specifically, the labeling effort was reduced to 10% of the entire dataset in all three tasks.

**Conclusions:**

The set of preferred similar terms that are relevant to a chart review task can be learned by leveraging the medical context of the task.

**Supplementary Information:**

The online version contains supplementary material available at 10.1186/s12911-021-01724-2.

## Background

In a clinical chart review task [1], a clinically knowledgeable person (e.g., physician, medical student, or nurse) combs through electronic medical records (EMRs) [2–4] for specific data of interest. Chart reviews are time-consuming and costly because a patient’s chart may be composed of hundreds of clinical notes. Various automated approaches have been developed to improve the efficiency of chart reviews. A particularly promising information retrieval (IR) method to assist with chart reviews is query expansion [5–8]. This method expands the original search terms into a set of similar terms and, subsequently, returns medical notes that contain at least one of the expanded terms. In addition, these similar terms can be applied to highlight text within a note and assist the reviewer to identify the important snippets of text quickly [9–14].

Chart reviews are relied upon to answer a wide range of questions—from determining the current stage of cancer for a particular patient to identifying which drugs appear to be most ordered for the treatment of seizures. These different chart review tasks can be assisted by query expansion methods; however, given the range of chart review tasks that derive from a single search term, a static set of similar terms is not appropriate for all tasks. Rather, the set of similar terms should adjust based on the context of the chart review task. For example, a reviewer looking for an epilepsy diagnosis likely cares more about EEG results, while a reviewer looking at medications for treating epilepsy likely cares more about indications of the drug Keppra. Therefore, the set of similar terms should dynamically adjust based on the task and context of the review.

To date, natural language processing methods for term similarity, such as word2vec [15, 16] and more recently Bidirectional Encoder Representations from Transformers (BERT) [17], provide embeddings to capture term similarity that can be used to recommend terms for expansion. For example, these methods now support dynamic query refinement in the Google search engine [17, 18]. Importantly, the similarity between two words within an embedding depends on the training data set used to build the embedding[19], as well as the data set used to fine-tune the model (e.g., refining BERT into BioBERT [20]). Thus, as the training or finetuning data set is changed, the set of expansion terms similarly will change.

While word2vec model training and finetuning activities modify word similarities according to textual relationships, there are a number of ways that clinical documentation can be influenced by factors not explicitly documented in the text. For example, word choice can be modified by a number of factors, including, but not limited to, who authored the note, the section in which the word is documented, or the age of the patient. Similarly, when reviewing charts, these different usages impact the information needed for a chart review. In this research, we investigate how such contextual information can be leveraged to modify term similarity for chart review tasks.

In this paper, we introduce a **medical-context vector space**, which corresponds to a collection of the usage frequencies of clinical terms in various real-world medical situations, to identify task-appropriate similar terms. We evaluate the medical-context vector space for prediction of preferred similar terms in chart review tasks for acute myocardial infarction (AMI), Crohn’s disease, and diabetes. Each of these tasks is notable in that they consist of complex requirements for identifying similar terms for chart reviews, including terms for relevant diagnoses, medications, findings, and history. Additional file 1: Table A, Table B, and Table C demonstrate the 10 most similar terms for "Crohn," "Acute Myocardial Infarction (AMI)," and "Diabetes." It can be seen that there are similar terms in common across the various medical contexts, as well as specific similar terms for certain medical contexts. For example, as shown in Additional file 1: Table A, “ileitis” and “ileum” are commonly used similar terms for “Crohn”, but “pancolitis" is only used in the outpatient-visit note types from the gastroenterology department, which implies that the system will recommend "pancolitis" to users only when they focus on reviewing a specific note type from a certain department.

## Methods

### Medical-context vector space

**Fig. 1:**
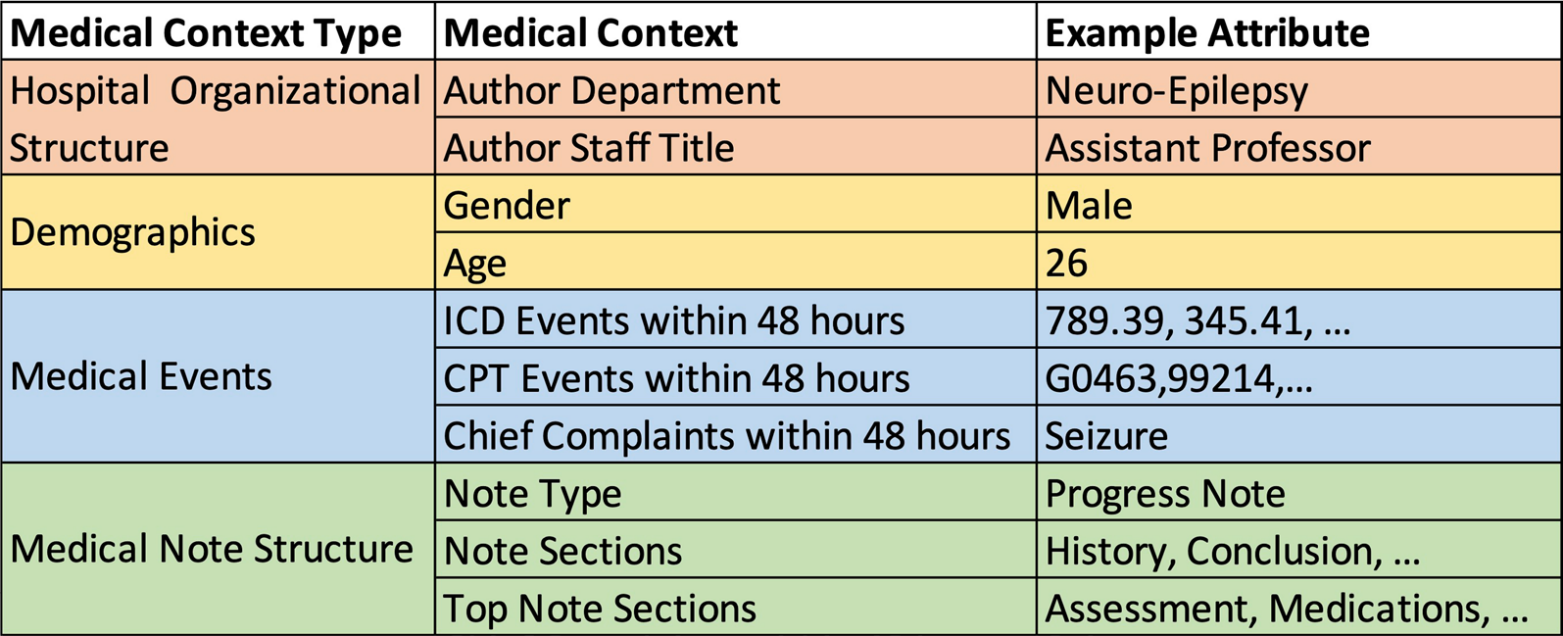
The medical context for an example clinical note

To orient the reader, we provide a running example in Fig. 1, which depicts the medical context associated with a fictitious medical note. The note was created for a 26-year-old male patient by a physician in the Neuro-Epilepsy Department. The *Medical Context Type* refers to the general context of a term’s usage, the *Medical Context* refers to a specific type of data in the context, and the *Attribute* refers to the specific value. Our objective is to capture information regarding how terms are used in different medical contexts.

We identified four types of usage context types resulting in ten specific contexts from the EMR system [21] of Vanderbilt University Medical Center (VUMC):

1. **Hospital Organizational Structure.** The role and speciality of the note’s author (i.e., job titles and departments) based on the hospital’s organizational structure.
2. **Medical Events.** The documented diagnoses and procedures of a patient, including ICD-9/10 codes, CPT codes, and Emergency Department chief complaints that are documented around the time the note is written.
3. **Demographics.** Patient gender (male, female, and unknown) and age (quantized into ten-year bins).
4. **Medical Note Structure.** Clinical note types and sections.

**Fig. 2:**
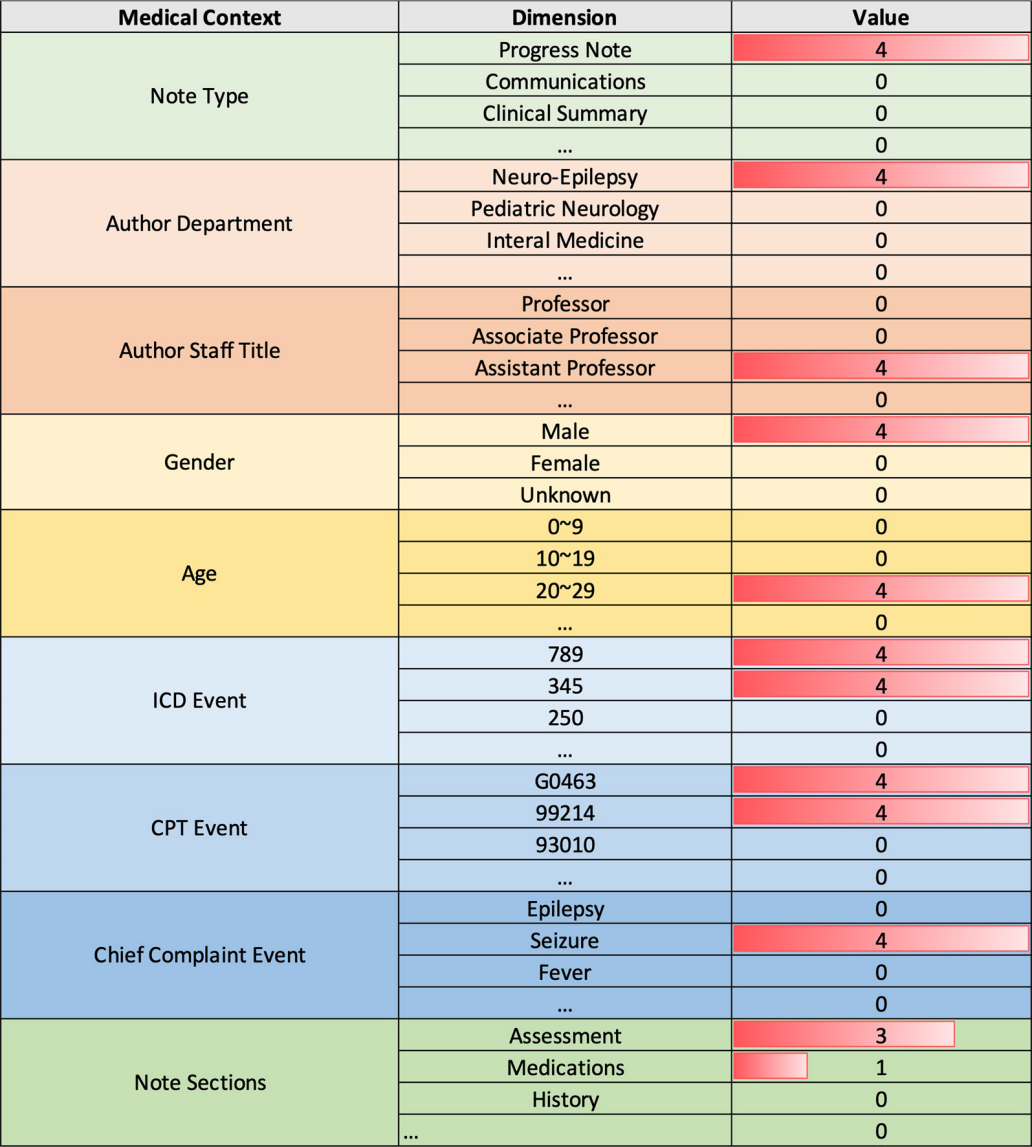
The medical-context counts of *EEG* according to their context in the example note in Fig. 1

**Fig. 3:**
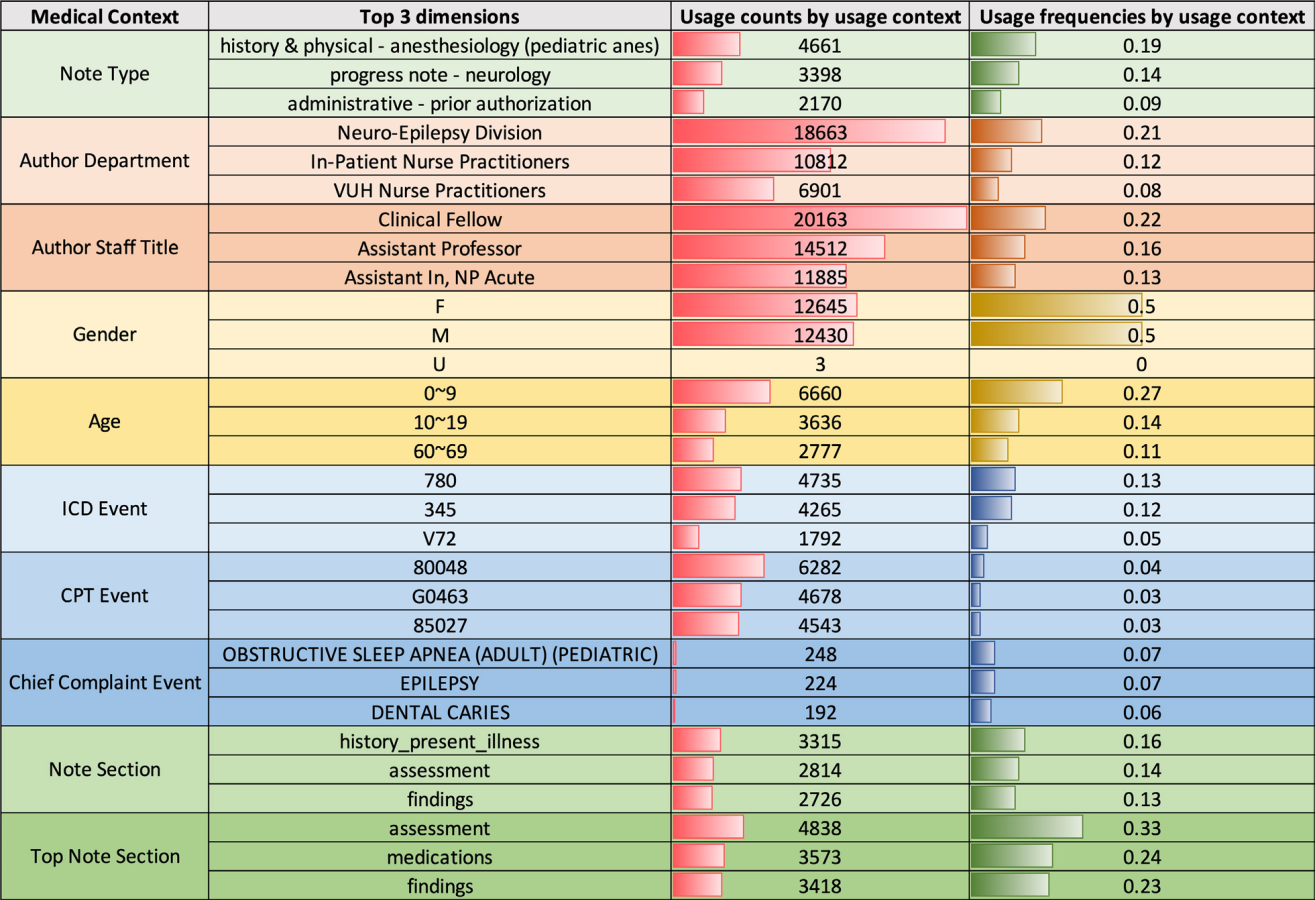
The top 3 dimensions in each medical context for *EEG*

These contexts represent commonly used descriptors of patient care and can be used to infer non-textual information regarding how terms are used in different situations. Leveraging this structure, we build the medical-context vector space through the following steps:

1. **Preprocessing**: First, we extract a subset of notes from the EMR system (e.g., all medical notes created in the year 2016). For each medical note and context, we extract the associated attribute values (as shown in Fig. 2) and filter out stop words (e.g., “a” and “of”) and single-character words.
2. **Initialization:** We define ten medical contexts $C=\left\{{C}_{1},C,.., {C}_{10}\right\}$ as shown in Table 1. For each clinical term ***w***, we initialize its medical-context vector to all zeros:
  $${u}_{w}=\{{u}_{c1}\left(w\right), { u}_{c2}\left(w\right), \dots , { u}_{c10}(w)\}=\left\{{\overrightarrow{0}}_{c1},{\overrightarrow{0}}_{c2},\dots , {\overrightarrow{0}}_{c10}\right\}$$
3. **Accumulation:** For each word, we increment its medical-context vector based on the occurrence of the word in each context. For example, in Fig. 2, we add four to the *Neuro-Epilepsy* dimension of the *author’s department* medical context in the medical-context vector of *EEG* if the author uses EEG four times. This step is repeated for each note.
4. **Normalization:** Next, as shown in Fig. 3, we normalize the counts of clinical terms in each context into medical-context proportions [0.0, 1.0] (i.e., the medical context vectors). At the end of this process, each clinical term is represented as a medical-context vector that consists of its normalized frequencies in each medical context.

**Table 1:** The dimensions for clinical terms in each medical context

Context type	Medical context	Dimensions
Hospital organizational structure	Departments	258
	Staff	158
Medical events	CPT events	6537
	ICD events	957
	Chief complaint events	11,595
Demographics	Age	10
	Gender	3
Medical note structure	Note type	1514
	Note section	61
	Top note section	5

The medical-context vector represents how a term is used within a medical situation. We define the medical-context similarity of two clinical terms ${w}_{i}$ and ${w}_{j}$ in the medical context ${C}_{k}$ as the cosine similarity of their medical-context vectors:

$${S}_{{c}_{k}}\left({w}_{i},{w}_{j}\right)=\frac{{u}_{{c}_{k}}({w}_{i})\cdot {u}_{{c}_{k}}({w}_{j})}{\parallel {u}_{{c}_{k}}({w}_{i})\parallel \times \parallel {u}_{{c}_{k}}({w}_{j})\parallel }$$

**Fig. 4:**
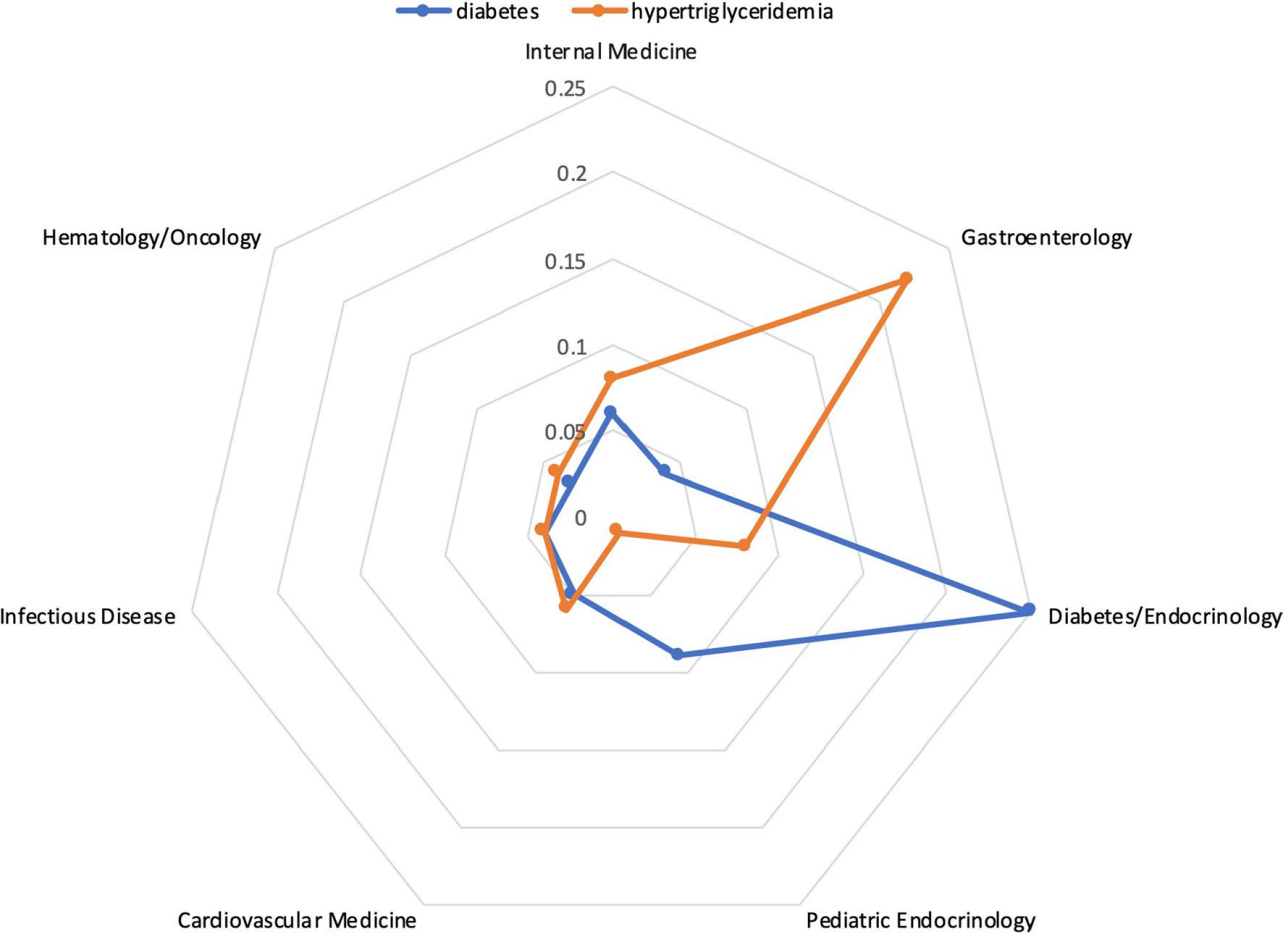
The medical-context vectors for *diabetes* and *hypertriglyceridemia* in the *Department* medical context

The similarity of two clinically similar terms in a medical context provides intuition into their semantic relationships. For example, as shown in Fig. 4, in the department medical context, the cosine similarity of *diabetes* and *hypertriglyceridemia* is 0.56, which suggests that they are moderately similar in the department medical context, while other terms have a better similarity (e.g., hypothyroidism) in that context. For further illustration, Additional file 1: Table A, Table B and Table C report on the top similar terms in different medical contexts.

We define the medical-context similarity vector of two clinical terms ${w}_{i}$ and ${w}_{j}$ as a vector containing the medical-context similarities across all medical contexts. Each index of the vector is equal to the cosine similarity of each term’s medical-context vector for one specific context:

$$S\left({w}_{i},{w}_{j}\right)=\{{S}_{c1}\left({w}_{i}, {w}_{j}\right), {S}_{c2}\left({w}_{i},{w}_{j}\right), \dots , {S}_{c10}({w}_{i},{w}_{j})\}$$

**Fig. 5:**
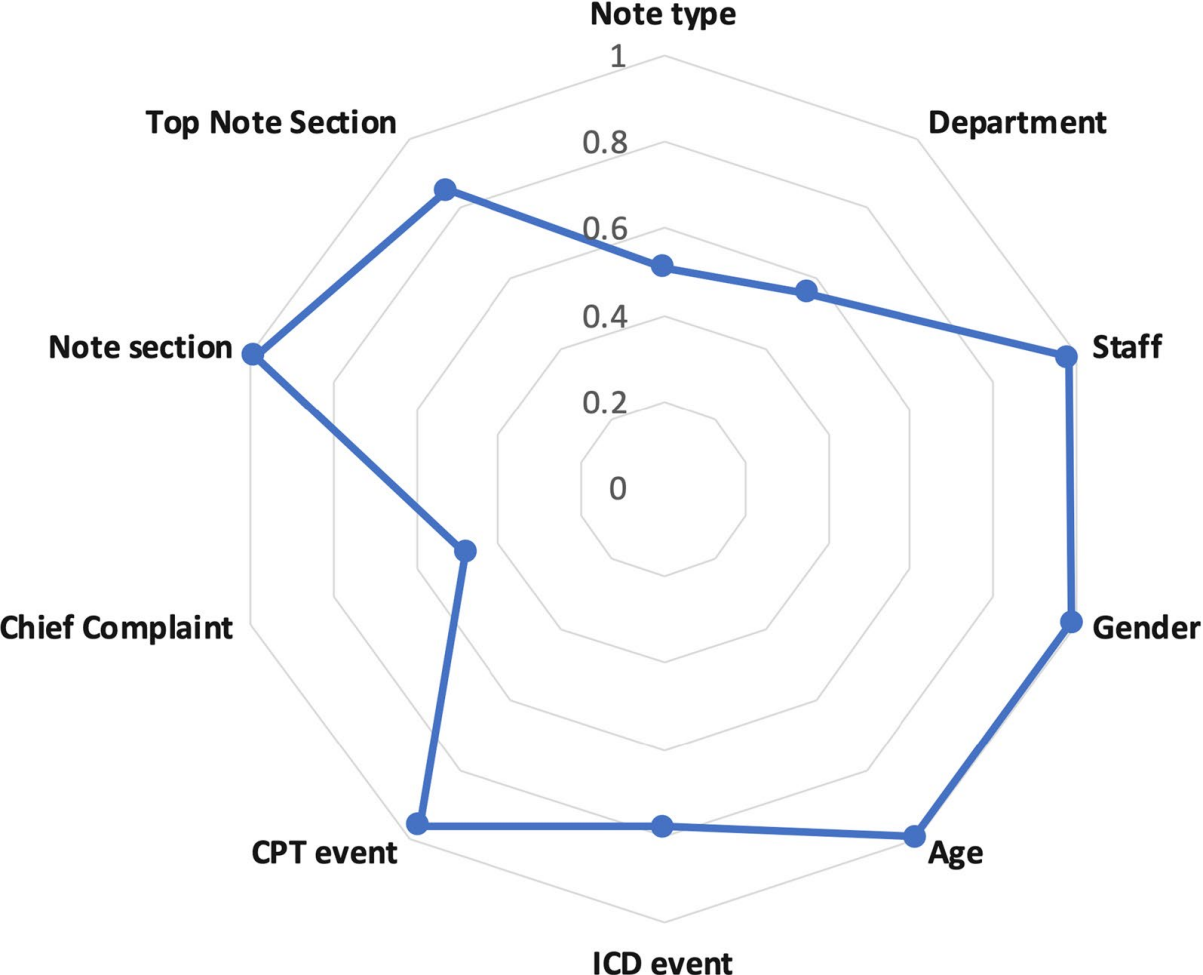
The medical-context similarities of *diabetes* and *hypertriglyceridemia* in all medical contexts

The medical-context similarity vector of two clinical terms represents their relationships across all medical contexts. For example, Fig. 5 shows the medical-context similarity vector of *diabetes* and *hypertriglyceridemia*. It can be seen that their similarity in the *Note Type*, *Author Department,* and *Chief Complaints* contexts are much lower than in other contexts. Therefore, if a reviewer prefers terms that have a similar distribution of medical-context frequencies as *diabetes* in the *Note Type*, *Author Department,* and *Chief Complaints* contexts, then the reviewer may not prefer *hypertriglyceridemia*.

Medical-context similarity vectors provide a weighted vector space, which can be used to identify which similar terms are preferred for a specific chart review task. Thus, instead of providing chart reviewers with a static set of similar terms, the set can be adjusted as information is collected about the context of a task. This adjustment can be done in an online learning style where (i) a chart reviewer first inputs a keyword, (ii) is presented with a list of ranked similar terms, and (iii) the reviewer then starts the chart review task, in which the goal is to highlight text in notes that are evidence for answering clinical questions. Given the iteratively-gathered highlighted text as input, a supervised machine learning model for term prediction is iteratively trained after each input to capture the contexts that the reviewer deems to be most important. The trained model is then applied to recommend similar terms.

### Evaluation

#### Medical-context vector space

We collected notes from the VUMC EMR generated between January 1, 2016, and January 2, 2017. The medical contexts were distributed across a set of dimensions as follows (and shown in Table 1):

1. **Hospital Organizational Structure.** 258 departments and 158 types of staff;
2. **Medical Events.** 957 ICD-9 codes, 6,537 CPT codes and 11,595 chief complaints in free-text format;
3. **Demographics.** Three patient genders (male, female and unknown) and ten age ranges (quantized into ten-year bins up to 100, after which all ages were represented as 100 +);
4. **Medical Note Structure.** 1,514 note types; 61 note sections (defined by the headers as determined by the SecTag method [22]). Five sections (“assessment,” “findings,” “family medical history,” “medications,” and “problem list”) contain the most important information in a chart review task based on our discussions with the medical researchers.

#### Datasets

We created three **evaluation datasets** associated with chart review tasks (Table 2):

1. Acute Myocardial Infarction Note Relevance (referred to as the **AMI project**). This task requires researchers to highlight any portion of a note that contains references to diagnoses, medications, procedures, or symptoms of AMI.
2. Crohn’s Anti-TNF Responsiveness (referred to as the **Crohn’s project**). This task requires researchers to review and highlight text describing whether a patient with Crohn's disease was clinically responsive to anti-TNF medication.
3. Pediatric Diabetes Note Barriers (referred to as the **Diabetes project**). This task requires researchers to review a list of medical notes, highlight and label portions of the notes that may be related to barriers in the documentation of diabetes plans.

**Table 2:** Chart review tasks defined for the evaluation

Chart review task	Topic word	Patients	Notes
Acute myocardial infarction	AMI	152	200
Crohn’s anti-TNF Responsiveness	Crohn	983	437,993
pediatric diabetes note barriers	Diabetes	76	210

All of these chart review tasks were deployed in the Vanderbilt’s Pybossa crowdsourcing platform [23], and reviewed by chart reviewers who have sufficient medical knowledge. We recruited medical researchers from different disciplines of VUMC, including professors, nurses, and medical students who passed a pre-citification of medical knowledge related to the chart review tasks.

In each of the chart review tasks, the researchers searched and reviewed medical notes to identify and highlight important text snippets for the task. Given the medical notes *D* of a chart review task *T*, we define the highlighted count *H* of a clinical term $w$ as the total number of times *w* is highlighted across all documents in *D*:

$$H\left(w|D\right)= \sum_{{d}_{j}\in D}H\left(w|{d}_{j}\right)$$

#### Experimental design

We assessed the capabilities of the medical-context vector space and standard word2vec methods by evaluating the extent to which the methods identified the terms that chart reviewers would highlight. Specifically, the term prediction supervised machine learning model is provided highlighted and non-highlighted text as labeled input along with either the medical-context vectors or word2vec vectors, and then predicts if terms will be highlighted. The two hypotheses driving this experiment are: (i) if a term is relevant to a task, then the term should be highlighted by the chart reviewer, and (ii) the terms that are highlighted the most often should be preferred (i.e., predicted by the model) at a higher frequency than non-preferred terms. The experimental design is as follows.

For each chart review task, a topic word K is chosen as the most important keyword of the research goal (e.g., *diabetes* is a topic word of the research task *Pediatric Diabetes Note Barriers Problem*) and serves as the basis for a similar term generator. Table 2 presents the topic word of each chart review task.

We define the similar terms that might be preferred by the researchers of a chart review task as the **candidate semantic set.** A candidate semantic set ${W}_{s}$ can be provided by any existing similar term generator, such as EMR-based word2vec embeddings [6, 14, 24, 25], or the EMR-subsets method. A candidate set is used instead of all possible words in the vocabulary as a means to limit the search space.

We define the **semantic preference** of a chart review task as a subset of preferred similar terms and a subset of non-preferred similar terms from the candidate semantic set. A **semantic preference prediction** task is formulated as a supervised machine learning task, in which a model learns the semantic preference from a small set of preferred similar terms and non-preferred similar terms (i.e., the training label set). The features of a similar term *w* are its medical-context similarity vector based on the topic word *K*. The label of a similar term is based on its highlighted count in the chart review task and a given importance cutoff *I*. If the highlighted count of a similar term $\mathrm{w}\in {W}_{s}$ is greater than *I*, we label it as an important term (i.e., label = 1); otherwise, we label it as a non-important term (i.e., label = 0).

**Fig. 6:**
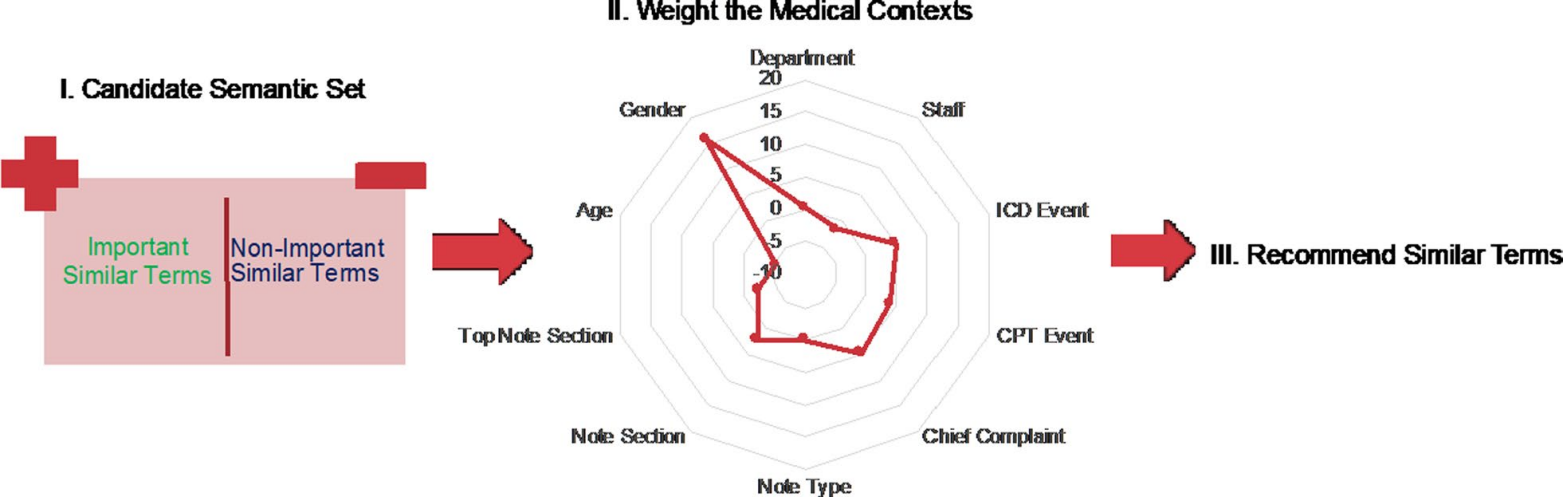
The workflow for learning and recommending clinically similar terms by reweighting medical-context similarity vectors

Figure 6 shows an example application of the medical-context vector space to predict the preferred similar terms of reviewers in a chart review task. A classifier based on logistic regression is trained to weight each medical context and obtain the weights of medical context as ${W}_{c}=\{{W}_{c1},{W}_{c2},\dots ,{W}_{c10}\}$ with a given threshold $I$. Given the medical-context similarity vector $\{{S}_{c1}\left(K,w\right), {S}_{c2}\left(K,w\right), \dots ,{S}_{c10}\left(K,w\right)\}$ of an unlabeled similar term *t*, the classifier then predicts if an unlabeled similar term *t* is a preferred similar term of the reviewer and will be highlighted.

### Semantic preference prediction evaluation

This semantic preference prediction task evaluation was performed in the following manner:

1. Given an evaluation data set, we first generate a candidate semantic set ${W}_{s}$ for its topic word using an existing similar term generator.
2. Given the candidate semantic set ${W}_{s}$, we construct a label set with an importance cutoff **I** = 1. If the highlighted count of a similar term ${w}_{i}\in {W}_{s}$ is greater than 1, we label it as an important term (i.e., label = 1); otherwise, we label it as a non-important term (i.e., label value equals 0). For each similar term ${w}_{i}$ in the candidate semantic set ${W}_{s}$, we generate its medical-context similarity vector $S({w}_{i}, K)$.
3. We train and evaluate a supervised machine learning model in the label set using ten-fold cross-validation. We evaluated three classifiers: (1) Logistic regression, (2) Random forest, and (3) Support vector machine. We measured the ROC (Receiver Operating Characteristic) curve and reported the AUROC (Area Under the ROC Curve). Other standard metrics (e.g., precision, recall, and F1 score) were considered; however, AUCROC was chosen for its ability to measure the balance of the true positive rate (TPR) and false positive rate (FPR).
4. We increase the importance cutoff *I* by 1 and repeat steps (2) and (3) until the number of important terms is less than 10 in the resulting label set. Based on the cross-validation model, we defined 10 as the minimum number of positive labels to ensure each test fold had at least one positive label.

We repeated this process with three similar term generators: (1) the EMR-subsets method [19], (2) the Complete EMR word2vec embedding [19], and (3) the Google News word2vec embedding [16]. The Complete EMR word2vec embedding and the Google News word2vec embedding are also used as baseline feature spaces to recommend similar terms. We use the two baseline embeddings to evaluate if the training data for the word embeddings significantly impacts performance.

### Learning curve evaluation

In a chart review task, the fewer labels required for learning the semantic preference, the earlier we can provide semantic support to reviewers. As such, we further assessed how the size of the training dataset influences the performance of the medical-context vector space. To perform this assessment, we rely on a learning curve analysis [26].

The learning curve analysis task evaluation was performed in the following manner:

1. Given an evaluation data set, we first generate a candidate semantic set ${W}_{s}$ using an existing similar term generator.
2. Given the candidate semantic set ${W}_{s}$, we constructed a label set with an importance cutoff *I*. When the highlighted count of a similar term ${w}_{i}\in {W}_{s}$ is greater than *I*, we label it as an important term (i.e., label = 1), otherwise, we label it as a non-important term (i.e., label value equals 0). For each similar term ${w}_{si}$ in the candidate semantic set, we generate its medical-context similarity vector $S({w}_{i}, K)$.
3. Given the label set, we set *x* to 1% of the data points as the training set and the remaining 99% as the test set.
4. We train a supervised machine learning model with the training set and evaluate its AUROC with the test set. Repeat step (3) and (4) 100 times and measure the AUROC.
5. Next, we increase *x* by 1% and repeat step (3) and (4) until x is greater than 90%.
6. Finally, we increase the importance cutoff *I* and repeat step (2) to (5) until the number of important terms is less than 10 in the resulting label set.

We repeat this process with the similar term generators used in the Semantic Preference Prediction Experiment.

### Interpretable feature space experiment

Constructing interpretable feature space is essential for medical applications [27, 28], especially chart review tasks. Thus, we assessed the potential of the medical-context vector space for providing an interpretable feature space. We applied the binary logistic regression (Eq. 1) to analyze the impacts of medical contexts to reviewers’ semantic preference of the three chart review tasks (Table 2) for a term *w* and the topic word *K*, and interpreted the meanings of the weights of each medical context.

1 $$ln\left(\frac{P\left(Preferred\right)}{P\left(Non{\text{-}}preferred\right)}\right)= Intercept + \sum_{i=1}^{10}{C}_{i}*{S}_{{c}_{i}}(w, K)$$

## Results

### Distribution of terms

**Fig. 7:**
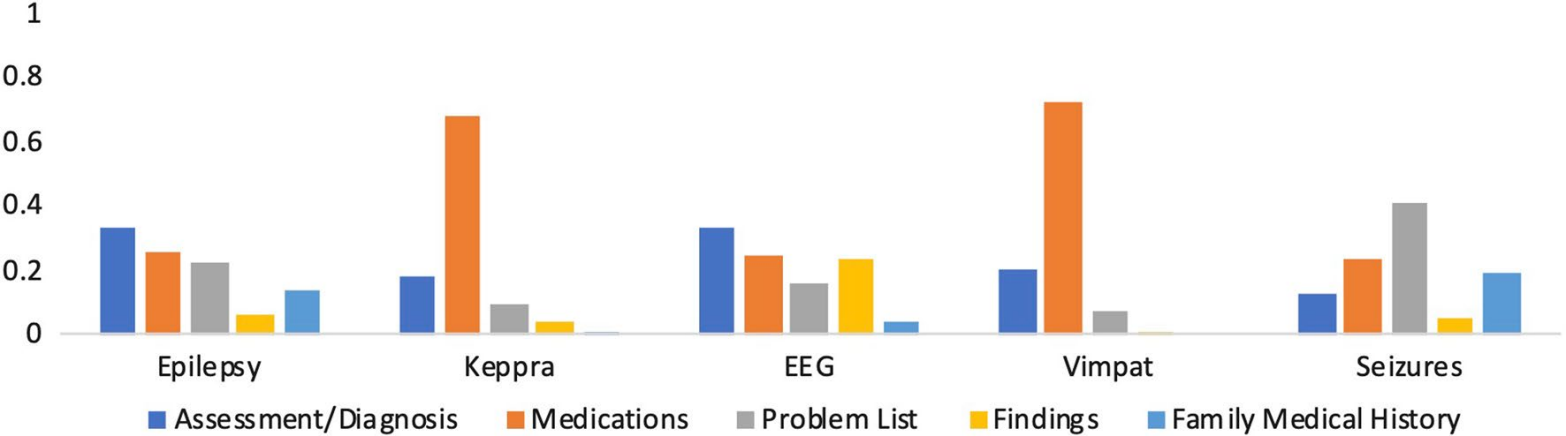
The proportion of similar terms for *epilepsy* in note sections

Figure 7 shows the distribution of the clinical terms (*Keppra*, *EEG*, *seizures*, *epilepsy,* and *Vimpat*) across the note sections. It can be seen that *EEG* is frequently used in the *Assessment/Diagnosis* section, while *Keppra* is more frequently used in the *Medications* section.

### Comparison to BERT

We compare the medical-context vector’s performance to two fine-tuned BERT models: (i) a BERT model fine-tuned by the approximately four million de-identified prescription notes from the VUMC EMR (which we refer to as the DrugBERT model), and (ii) the pre-trained BioBERT model. We provide the phrase “*Keppra medication”* to these models, in which *medication* is the contextual information to help the models contextualize similar terms for Keppra. For the medical-context vector, the phrase Keppra medication is converted into a context vector. For all methods, we find the ten most similar terms.

Table 3 presents the similar terms for *Keppra* from the models. The result shows that the DrugBERT and BioBERT model provides mostly typos of *Keppra* (e.g., *keppr*) as the top similar terms of "Keppra." However, in a clinical chart review task, given the phase *Keppra medication*, it is more likely the reviewers consider drugs other than *Keppra* as its top similar terms.

**Table 3:** Similar terms for *Keppra* based on the fine-tuned BERT models and the medical-context vector space

Similarity rank	DrugBERT	BioBERT	Medical-context vector space
1	keprra	keppr	depakote
2	keppr	onkeppra	vimpat
3	keppera	kepppra	trilepatal
4	keprpa	keppraxr	valproic
5	sezure	keprra	phenobarbital
6	gabatril	prnno	topiramate
7	sezire	ssri	gabatril
8	sizure	andmri	lamictal
9	seizre	najib	fosphenytoin
10	equetro	nimotop	zonisamide

### Semantic preference prediction

Table 4 shows the size of the candidate semantic set provided by the EMR-subsets method and the number of terms highlighted by reviewers. The table shows that for each project, reviewers highlighted different proportions of terms, demonstrating potential variability and challenges for recommending similar terms. In the remainder of this paper, we only show the results based on the candidate semantic sets provided by the EMR-subsets method and the baseline Complete EMR word2vec embedding because the Google New embedding resulted in similar results to the EMR embedding.

**Table 4:** The candidate semantic sets of the chart review tasks

Dataset	Candidate similar terms	Unique highlighted similar terms
AMI	1949	1414
Crohn	1204	438
Diabetes	1055	273

The medical-context vector space’s AUCROC outperformed all baseline word2vec embeddings in all evaluation datasets across all similar term generators. Tables 5, 6 and 7 provide the three example comparisons of the medical-context vector space and the baseline Complete EMR word2vec embeddings for three datasets. A one-sided Mann–Whitney U test indicated that the medical-context vector space statistically significantly outperformed the baseline Complete EMR word2vec embedding.

**Table 5:** Diabetes dataset average ROC AUROC scores with an importance cutoff of 10

Model	AUROC	
	Medical-context vector space features	word2vec features
Logistic regression	0.80*	0.58
Random forest	0.68*	0.54
Support vector machine	0.78*	0.57

*p < 0.05

**Table 6:** AMI dataset average ROC AUROC scores with an importance cutoff of 40

Model	AUROC	
	Medical-context vector space features	word2vec features
Logistic regression	0.80**	0.73
Random forest	0.75***	0.56
Support vector machine	0.75*	0.71

***p < 0.001, **p < 0.01, *p < 0.05

**Table 7:** Crohn dataset average ROC AUROC scores with an importance cutoff of 1

Model	AUROC	
	Medical-context vector space features	word2vec features
Logistic regression	0.79**	0.68
Random forest	0.80***	0.60
Support vector machine	0.79***	0.68

***p < 0.001, **p < 0.01, *p < 0.05

**Fig. 8:**
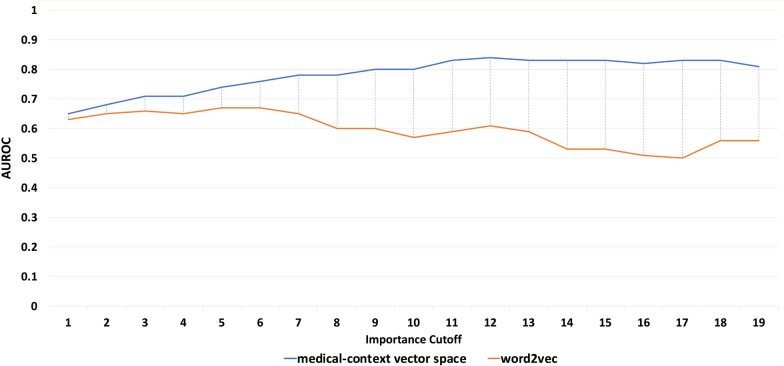
Average AUROC achieved by the logistic regression classifier for the diabetes dataset

Figure 8 shows the result of the Semantic Preference Prediction evaluation using the Diabetes dataset and the candidate semantic set generated by the EMR-subsets method [19].

### Learning curve analysis

**Fig. 9:**
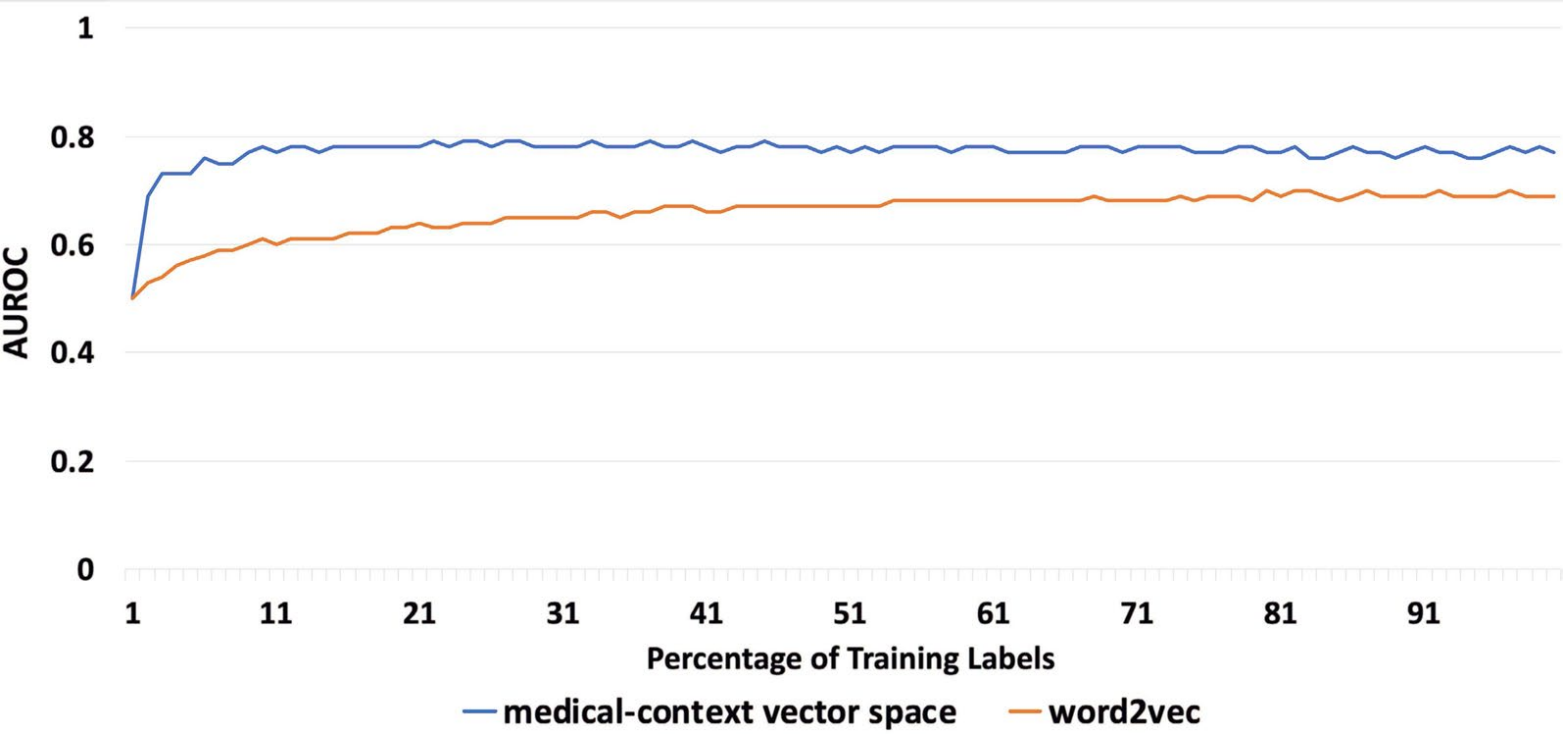
Average AUROC with different training dataset size for the Crohn’s disease dataset (importance cutoff 1)

As shown in Fig. 9, the medical-context vector space outperformed the EMR-based word2vec embedding regardless of the size of the training data set. It can be seen that the medical-context vector space significantly reduces the number of required labels for learning the semantic preference. For example, as shown in Fig. 9, with only 1% of the label set, the medical-context vector space reached an AUROC of 0.7 while the baseline Complete EMR word2vec embedding only achieved 0.5. When using 10% of the labels, the medical-context vector space and word2vect achieved an AUROC of 0.78 and 0.60, respectively.

### Interpretable feature space

As shown in Table 8, the *Chief Complaint* medical context has a significant positive impact on reviewers’ semantic preference, which means the clinical terms that are similar in describing the same chief complaint of a chart review task are preferred by the reviewers. It is interesting that the *Gender* context had the highest significant positive impact on the semantic preference of the AMI chart review task. Since the topic word *AMI* likely has little relevance with respect to gender, terms highly relevant to gender were not preferred by the reviewers.

**Table 8:** The impact of medical contexts on reviewers’ preference in AMI task

Index	Context	Coefficient		
		AMI	Crohn’s	Diabetes
1	Intercept	− 16.16***	− 13.82***	− 22.04
2	Department	0.58	0.42	0.33
3	Staff	1.90	0.64	− 1.49
4	ICD event	− 2.94**	2.18**	4.89
5	CPT event	0.37	1.35*	4.10
6	Chief complaint	5.75***	7.24***	4.93***
7	Note type	5.70***	− 1.92*	− 0.05
8	Note section	2.00***	− 0.23	2.37**
9	Top five note sections	1.43	1.37*	− 2.54
10	Age	0.37	2.65***	− 5.41***
11	Gender	8.85***	8.03***	15.78

***p < 0.001, **p < 0.01, *p < 0.05, one-tailed

## Discussion

This paper presents a novel vector space model, the medical-context vector space, to identify similar terms to support chart reviews. The medical-context vector space is a collection of normalized-frequencies of clinical terms in different medical contexts, which provide information on the relationships between clinical terms. We evaluated the medical-context vector space for predicting the preferred similar terms of reviewers in three chart review tasks. The results show that the medical-context vector space efficiently learned the preferred similar terms of reviewers and outperformed the baseline word2vec embedding in all three chart review tasks as measured with the AUROC metric. Additionally, the medical-context vector space significantly reduced the number of labels (e.g., from thousands of labels to tens of labels) required to learn and predict the preferred similar terms of reviewers.

There are several possible reasons why the medical-context vector space outperformed the baseline methods. First, the feature space provided by the medical-context vector space is much smaller than the feature space provided by the word2vec embedding (i.e., 10 dimensions vs. 100 dimensions of the Complete EMR word2vec embedding). Second, the feature space provided by the medical-context vector space is more capable of capturing relationships between terms induced by external, non-textual forces. For instance, context such as the chief complaint, the author’s department and the patient’s age influence the terms a chart reviewer prefers for a given task, yet these factors are not captured in traditional word embedding models. Third, the construction of medical contexts (e.g., note sections), and counting the frequency of words in those contexts, implicitly captures relationships between terms in structured ways that otherwise would be difficult to extract based on the text alone.

The medical-context vector adjusts to the reviewer’s desired semantics by eliciting reviewer input. Previous research has similarly demonstrated that clinical natural language processing models (e.g., word sense disambiguation) can be trained by asking experts to provide labeled instances [29, 30]. This iterative process allows the medical-context vector to essentially be fine-tuned for the specific task. While other fine-tuning methods have been proposed and shown to be successful using text, the fine-tuning process used in this work relies on the non-textual contexts that are encoded. This explicit encoding of context allows for rapid learning of the reviewer’s preference, as demonstrated by the number of labels needed from reviewers.

Fine-tuned BERT models, such as the DrugBERT and BioBERT, can be tuned for a specific chart review task. However, this study shows that the resulting embeddings recommend terms in different ways than the medical-context vector. For example, when looking for similar terms of a seizure drug, BERT fine-tuned models recommend typos or misspellings, while the medical-context vector recommends other drugs with the same clinical purpose. Thus, while BERT and fine-tuned BERT can be useful for expanding terms for clinical chart review tasks, the medical-context vector fills in an essential technological gap when identifying similar terms based on the context in which terms are used.

In this study, three machine learning methods (namely, logistic regression, random forest, and support vector machines) were evaluated to determine how well they predict the preferred terms for clinically knowledgeable reviewers.

All three machine learning models attained better performance when using the medical-context vector space compared to the word2vec embeddings. In a follow-up pilot study, several clinical researchers were invited to test a prototype user interface (Additional file 1: Figure D). Their feedback suggested that the logistic regression method might be preferred by clinical researchers because the results are both accurate and easily interpretable by examining the weights of the logistic coefficients).

By contrast, methods based on random forests and support vector machines can be more difficult to interpret due to the complexity of the models. Specifically, random forests learn a list of features, which creates subgroups of variables, and then builds an ensemble over the subgroups. Support vector machines identify a decision surface in a feature space that is higher in dimensionality than the original system to separate different classes.

There are several limitations of this study that highlight opportunities for future research. First, when building the medical-context vector space, we limited the time range used to build the medical event context of a note to 48 h (i.e., a chief complaint had to be within 48 h of when the note was written). It is unknown if different time ranges would impact these findings. Second, in this study, we focused on ten medical contexts when building the medical-context vector space, but there are clearly others that could be considered. Third, this pilot study indicated that certain medical contexts (e.g., the “Chief Complaint” medical context) have a significant impact on reviewers’ semantic preferences in a chart review task, but it is necessary to survey reviewers to learn why they prefer such terms.

To refine this system, the vector space could be enhanced in several ways. First, domain knowledge could be introduced by adding more knowledge-based dimensions, such as a laboratory result dimension, which would be oriented to capture how providers use words when describing test values. Second, the system could be extended by inviting clinical researchers to identify the important medical contexts. As shown in Additional file 1: Figure D, a clinical researcher selects words from a candidate word list (left column of the UI) and drag each word into the positive (negative) area if they are preferred (or not). The system will learn the preferred contexts of clinical researchers and identify the most important medical contexts.

The vector space method can be put into practice and further tested in our existing EMR search engine (Additional file 1: Figure E) [19, 23]. The search engine takes a keyword as input which is expanded to a set of terms used for document retrieval based on the vector space.

## Conclusions

In this paper, we presented a novel vector space model, the medical-context vector space, to represent how clinical terms were used in varying medical situations. We evaluated the performance of the medical-context vector space in predicting the preferred similar terms of reviewers in three chart review tasks. The empirical findings show that the medical-context vector space achieved good performance and significantly outperforms baseline word2vec embeddings. Additionally, the medical-context vector space significantly reduced the number of labels required to learn and predict the preferred similar terms of reviewers. This research suggests that the medical-context vector space can better identify preferred similar terms based on non-textual features compared to traditional word embedding models.

## Supplementary Information

**Additional file 1.** Supplemental Appendix.

## Data Availability

The data for this study was based on the VUMC data repositories. The data are available within VUMC but restrictions are applied to the public availability of these data, and so are not publicly available. Data are however available from the corresponding author upon reasonable request and with the permission of VUMC.
