# Supplementary material for: Leveraging medical context to recommend semantically similar terms for chart reviews
Source: BMC Med Inform Decis Mak. 2021 Dec 18;21:353. doi: 10.1186/s12911-021-01724-2 (PMC8684266; doi:10.1186/s12911-021-01724-2)
Supplement: Supplementary file 1 — Additional file 1. Supplemental Appendix. [file 12911_2021_1724_MOESM1_ESM.docx]

**APPENDIX**

Table-A, Table B, and Table-C are relied upon as supplemental material in the Introduction and Methods sections of the main manuscript. These tables present the distribution of the ten most similar terms for the keywords “Crohn,” “Acute Myocardial Infarction (AMI),” and “Diabetes”, respectfully.

Figures D and E are relied upon as supplemental material in the Discussion section of the manuscript.  Figure D illustrates how the vector space method can be refined, while Figure E suggests how the vector space method can be put into practice.

| **Department** | **Staff** | **ICD Event** | **CPT Event** | **Age** | **Gender** | **Note Type** |
| --- | --- | --- | --- | --- | --- | --- |
| ileitis | lialda | ileitis | ileitis | ibd | lialda | ileitis |
| lialda | seton | pentasa | ileum | ileum | ibd | lialda |
| infliximab | ileoanal | ileum | lialda | ileitis | psoriatic | infliximab |
| pancolitis | ileocolic | ileocolonic | asacol | infliximab | colonoscopy | pancolitis |
| ileum | asacol | colestid | rowasa | perianal | mesalamine | colitis |
| cortifoam | remicade | ileocolic | humira | remicade | inflammatory | ileum |
| thiopurine | ileum | fistulizing | ileocolonic | lialda | remicade | canasa |
| ulcerative | aphthous | thiopurine | proctitis | mesalamine | volvulus | asacol |
| colitis | setons | ileocecal | cimzia | fistulous | flagyl | ulcerative |
| extraintestinal | pseudopolyp | aphthous | canasa | proctitis | ileocolic | proctitis |

**Table-A: The top 10 similar terms for “Crohn” in different medical contexts.**

| **Department** | **Staff** | **ICD Event** | **CPT Event** | **Age** | **Gender** | **Note Type** |
| --- | --- | --- | --- | --- | --- | --- |
| infarction | infarction | infarction | infarction | hypertensive | hypertensive | infarction |
| arteriosclerosis | arteriosclerosis | arteriosclerosis | stenting | infarction | angiographically | arteriosclerosis |
| cabg | stenting | cad | arteriosclerosis | troponins | anginal | coronary |
| stenting | cabg | coronary | hypertensive | pcwp | hpl | stenting |
| ptca | rca | cabg | coronary | oliguric | infarction | ptca |
| cypher | ptca | stenoses | stented | nyha | troponins | stented |
| coronary | cypher | stented | ptca | anginal | cad | rca |
| collaterals | cad | stenting | scintigraphically | cad | ards | collaterals |
| cad | collaterals | angina | stoke | stented | dyslipidemia | xience |
| cachexia | coronary | collaterals | collaterals | angina | mpi | circumflex |

**Table-B: The top 10 similar terms for “Acute Myocardial Infarction (AMI)” in different medical contexts.**

| **Department** | | **Staff** | **ICD Event** | **CPT Event** | | **Age** | **Gender** | **Note Type** |
| --- | --- | --- | --- | --- | --- | --- | --- | --- |
| hypothyroidism | mellitus | | mellitus | | mellitus | proteinuria | cholesterol | mellitus |
| mellitus | hypertriglyceridemia | | nonproliferative | | retinopathy | mellitus | dyslipidemia | ldl |
| hypoglycemic | nonproliferative | | retinopathy | | nonproliferative | iddm | disease | retinopathy |
| nocturia | tobacco | | endocrinologist | | fructosamine | hyperglycemia | hypertension | hypothyroidism |
| hyperthyroidism | morbid | | microalbuminuria | | cerebrovascular | hypothyroidism | hypoglycemia | hypoglycemic |
| fructosamine | polydipsia | | hypoglycemic | | hyperlipidemia | microalbuminuria | borderline | hypertension |
| microalbuminuria | gastroesophageal | | hypoglycemia | | hypercholesterolemia | nocturia | mellitus | hdl |
| hypogonadism | deficiency | | diabetic | | hypertriglyceridemia | cardiomyopathy | type | hypogonadism |
| nonproliferative | microalbuminuria | | ldl | | obese | cholesterol | anemia | hyperthyroidism |
| retinopathy | hypothyroidism | | type | | type | accident | hdl | endocrinologist |

**Table-C: The top 10 similar terms for “Diabetes” in different medical contexts.**


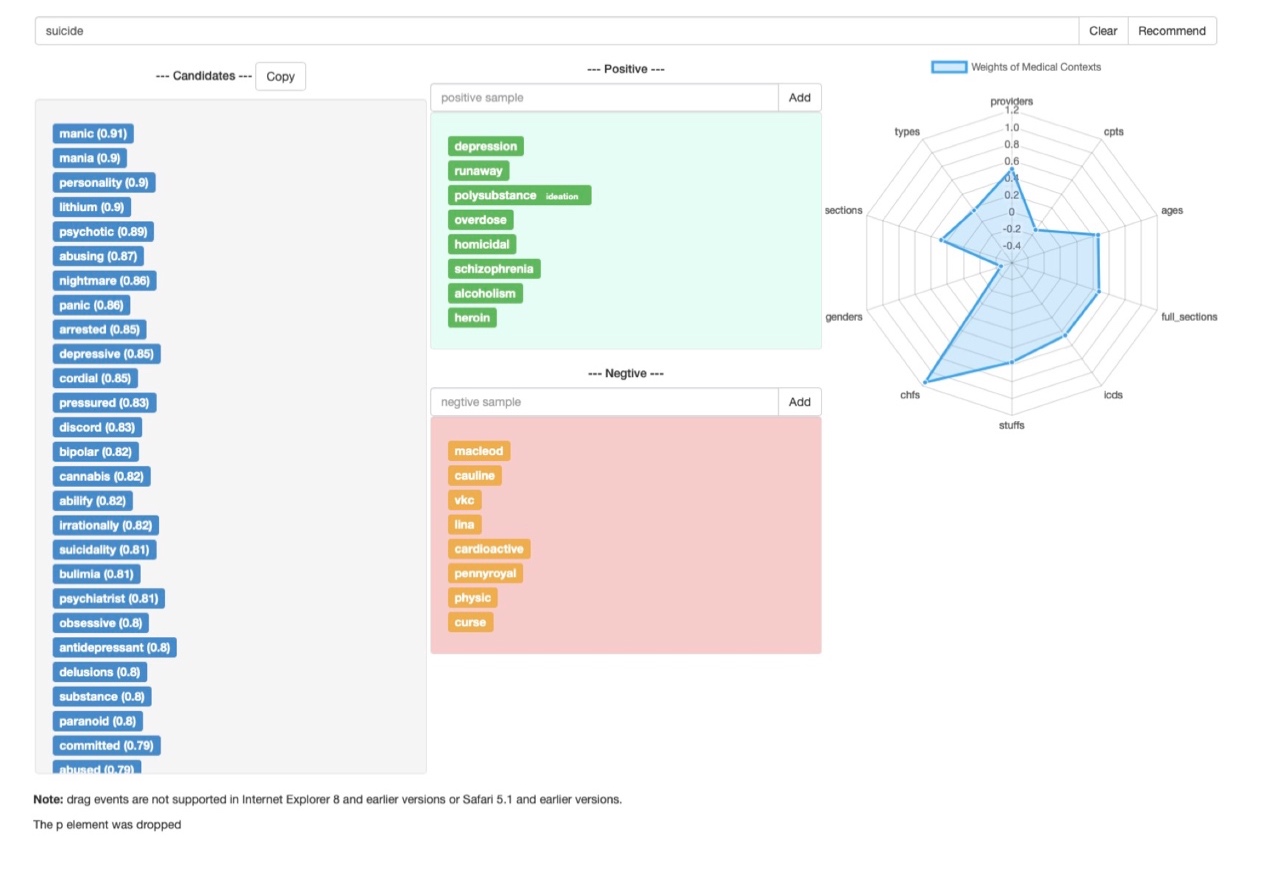


**Figure D. An UI for learning the relevance of medical contexts from the input of clinical researchers.**
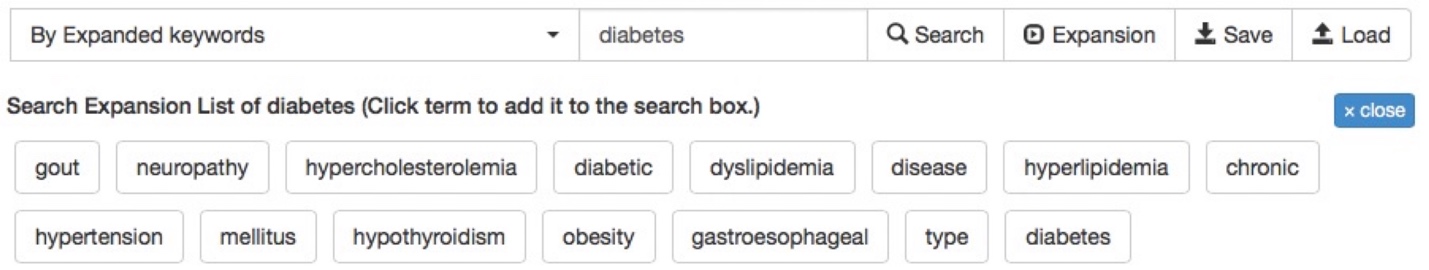


**Figure E. A prototype search engine interface with keyword recommendation based on the refined medical-context vector space.**
